# A genome wide association scan for (1,3;1,4)-β-glucan content in the grain of contemporary 2-row Spring and Winter barleys

**DOI:** 10.1186/1471-2164-15-907

**Published:** 2014-10-17

**Authors:** Kelly Houston, Joanne Russell, Miriam Schreiber, Claire Halpin, Helena Oakey, Jennifer M Washington, Allan Booth, Neil Shirley, Rachel A Burton, Geoffrey B Fincher, Robbie Waugh

**Affiliations:** The James Hutton Institute, Invergowrie, Dundee, DD2 5DA Scotland; ARC Centre of Excellence in Plant Cell Walls, School of Agriculture, Food & Wine, The University of Adelaide, Waite Campus, Glen Osmond, SA 5064 Australia; Division of Plant Sciences, University of Dundee at The James Hutton Institute, Invergowrie, Dundee, DD2 5DA Scotland

**Keywords:** Barley, (1,3;1,4)-β-glucan, Cell walls, GWAS, Soluble fibre

## Abstract

**Background:**

(1,3;1,4)-β-Glucan is an important component of the cell walls of barley grain as it affects processability during the production of alcoholic beverages and has significant human health benefits when consumed above recommended threshold levels. This leads to diametrically opposed quality requirements for different applications as low levels of (1,3;1,4)-β-glucan are required for brewing and distilling and high levels for positive impacts on human health.

**Results:**

We quantified grain (1,3;1,4)-β-glucan content in a collection of 399 2-row Spring-type, and 204 2-row Winter-type elite barley cultivars originating mainly from north western Europe. We combined these data with genotypic information derived using a 9 K Illumina iSelect SNP platform and subsequently carried out a Genome Wide Association Scan (GWAS). Statistical analysis accounting for residual genetic structure within the germplasm collection allowed us to identify significant associations between molecular markers and the phenotypic data. By anchoring the regions that contain these associations to the barley genome assembly we catalogued genes underlying the associations. Based on gene annotations and transcript abundance data we identified candidate genes.

**Conclusions:**

We show that a region of the genome on chromosome 2 containing a cluster of *CELLULOSE SYNTHASE-LIKE (Csl)* genes, including *CslF3*, *CslF4*, *CslF8*, *CslF10, CslF12 and CslH*, as well as a region on chromosome 1H containing *CslF9,* are associated with the phenotype in this germplasm. We also observed that several regions identified by GWAS contain glycoside hydrolases that are possibly involved in (1,3;1,4)-β-glucan breakdown, together with other genes that might participate in (1,3;1,4)-β-glucan synthesis, re-modelling or regulation. This analysis provides new opportunities for understanding the genes related to the regulation of (1,3;1,4)-β-glucan content in cereal grains.

**Electronic supplementary material:**

The online version of this article (doi:10.1186/1471-2164-15-907) contains supplementary material, which is available to authorized users.

## Background

Non-cellulosic polysaccharides from the cell walls of cereal grains are not digested by enzymes resident in the human small intestine, therefore they contribute to total dietary fibre intake [1]. Dietary fibre reduces the adverse social and personal impacts of serious human health conditions such as colorectal cancer, cardiovascular disease and type II diabetes [2], and the US Food and Drugs Administration (FDA) granted a claim that consumption of whole grain barley and barley–containing products reduces the risk of coronary heart disease, providing they comprise at least 0.75 grams of soluble fibre per 228 g serving [3–5]. However the use of barley as a food crop is not particularly common in western civilisations. There is an opportunity to change this and to simultaneously address the global health agenda, through the incorporation of novel barleys or barley products into a wide range of human food staples. This is particularly relevant in Northern Europe, where the climate and soils are well suited to barley production [6].

The effectiveness of non-cellulosic cell wall polysaccharides, including (1,3;1,4)-β-glucans, in improving health outcomes is related to their levels in grain, to their fine structures, and to their associated physicochemical properties. While most barley varieties contain 3-6% total fibre (compared to <1% in pearled rice, wheat, and triticale, and circa 4% in oats [7, 8], some contain more than 30% and have been marketed as health promoting super-foods (e.g. Sustagrain [9] and BarleyMax [10]). Within and between species, differences in (1,3;1,4)-β-glucan content can be due to both genetic variation and environmental conditions [11]. In barley, (1,3;1,4)-β-glucans are synthesized by members of the *CslF* and *CslH* gene families [12, 13]. The *CslF* gene family is comprised of ten members [14] and is part of the *CELLULOSE SYNTHASE* gene superfamily that is responsible for the synthesis of several plant cell wall polysaccharides [15]. Variation between individual members of the *CslF* and *CslH* gene families and/or the genes that regulate them (directly or indirectly) control the relative abundance and fine structure of (1,3;1,4)-β-glucans in both the grain and the rest of the plant [16]. Indeed, many of the very high (1,3;1,4)-β-glucan*-*containing barley accessions have mutations in starch biosynthetic genes, suggesting a regulatory link between starch and (1,3;1,4)-β-glucan content in cereal grains [17, 18]. In one class of mutants the link is believed to involve regulation of sugar nucleotide levels in the grain; with ADP-Glc being the glucose donor for starch biosynthesis, and UDP-Glc appearing to be the glucose donor supplying (1,3;1,4)-β-glucan synthesis [19]. Accordingly, over-expressing a *CslF* gene, under the control of an endosperm-specific promoter, resulted in an almost two-fold increase in (1,3;1,4)-β-glucan content in the transgenic grain [16]. Other grain constituents were largely unaffected, except for starch, which decreased dramatically in the high (1,3;1,4)-β-glucan lines. Grain composition in the model grass *Brachypodium distachyon* provides additional support for a regulatory link between starch and (1,3;1,4)-β-glucan synthesis [19]. There, endosperm cell walls are extremely thick, the (1,3;1,4)-β-glucan content of the grain is over 40% by weight and the starch content commensurately lower, at about 6% [20].

A more thorough understanding of the gene families that are responsible for both synthesising and hydrolysing (1,3;1,4)-β-glucan, and how they are regulated in barley and other cereal grains, is highly likely to facilitate innovative approaches to tailoring (1,3;1,4)-β-glucan content and its physicochemical properties to human health benefits. The opportunity for innovation is high, particularly because barley breeding has been traditionally targeted low grain (1,3;1,4)-β-glucan content to reduce viscosity and facilitate filtration during the brewing process. This trait has been the subject of many QTL mapping studies where low grain (1,3;1,4)-β-glucan content was the more desirable state [11, 21–23]. It seems likely therefore that high grain (1,3;1,4)-β-glucan content may have been intentionally bred out of elite malting quality varieties, with levels of variation in (1,3;1,4)-β-glucan content greater in varieties destined for (or consigned to) the non-malting sector. In support of this, [24] reported a range of grain (1,3;1,4)-β-glucan contents of 3.4% - 5.7% in a series of barley cultivars, while values of up to 13% have been reported for wild barley (*Hordeum spontaneum*) [25]. Since the FDA-backed health claim [3–5] was issued, the study of loci associated with grain (1,3;1,4)-β-glucan content has gained an additional dimension, fuelled by these diametrically opposed priorities regarding the preferred levels of grain (1,3;1,4)-β-glucan content depending on the end user market.

Here we report the results of a Genome Wide Association Scan (GWAS) of barley grain (1,3;1,4)-β-glucan content. We have previously used GWAS to identify genes regulating a range of traits in barley including grain density [26], flowering time [27], and row type [28]. However, compared to these traits, which were shown to be controlled by a small number of genes in the germplasm used, grain (1,3;1,4)-β-glucan content is a complex and so-called quantitative trait. Using a collection of both Spring- and Winter-type contemporary barley cultivars, largely originating from north-western Europe, combined with a densely populated SNP marker platform [27], we show that GWAS resolves previously identified QTL with increased precision, and highlights additional genetic regions and candidate genes for follow-up experiments.

## Results

### Grain (1,3;1,4)-β-glucan content

**Figure 1:**
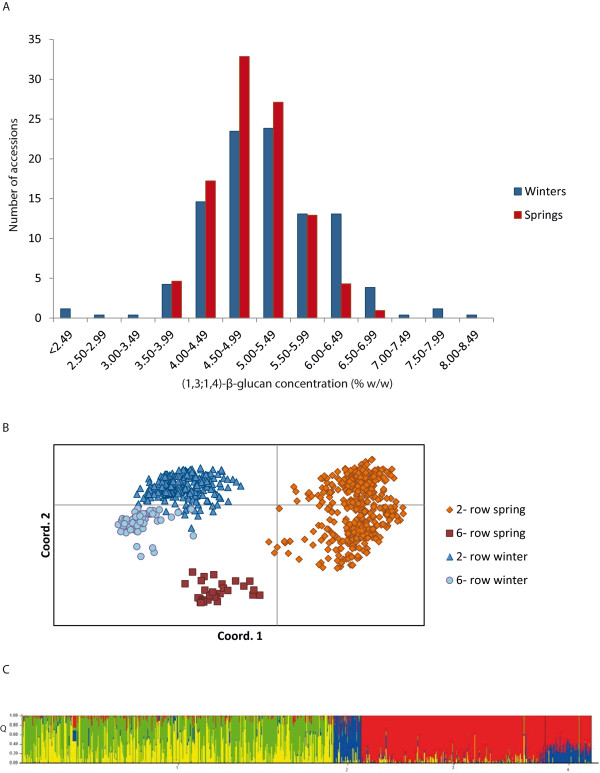
**Phenotypic and genotypic data used to carry out a genome wide association scan (GWAS). (A)** Mean grain (1,3;1,4)-β-glucan content for 204 Winter 2–row, and 399 Spring two – row elite barley lines. **(B)** A Principal Coordinates Analysis (PCoA) plot of the first two components of 9 K SNP iSelect genotyping data for766 elite Spring and Winter barley cultivars. This includes both row types for Spring and Winter cultivars **(C)** STRUCTURE bar plot for *K* = 4 grouped by flowering habit and row type based on 9 K SNP iSelect genotyping data for 766 elite Spring and Winter barley cultivars ordered by predetermined subpopulations. Subpopulation 1 = 2-row Springs, 2 = 6-row Springs, 3 = 2-row Winters, 4 = 6-row Winters. Q value represents proportion of ancestry to a given subpopulation.

The (1,3;1,4)-β-glucan values in the association/diversity panel ranged from 2.2% – 8.4% (Figure 1A). Correlations between true biological replicates for the Spring-type accessions were high (p = <0.001) and there were high levels of correlation between the two years for the Winter-type accessions (p = <0.001). In both sets of germplasm analysis of variance showed that significant variation occurred between accessions (p = <0.001), but not between assay batch order or date. The mean grain (1,3;1,4)-β-glucan contents for the Spring and Winter accessions were 4.95% and 5.10% respectively.

### Population structure

**Figure 2:**
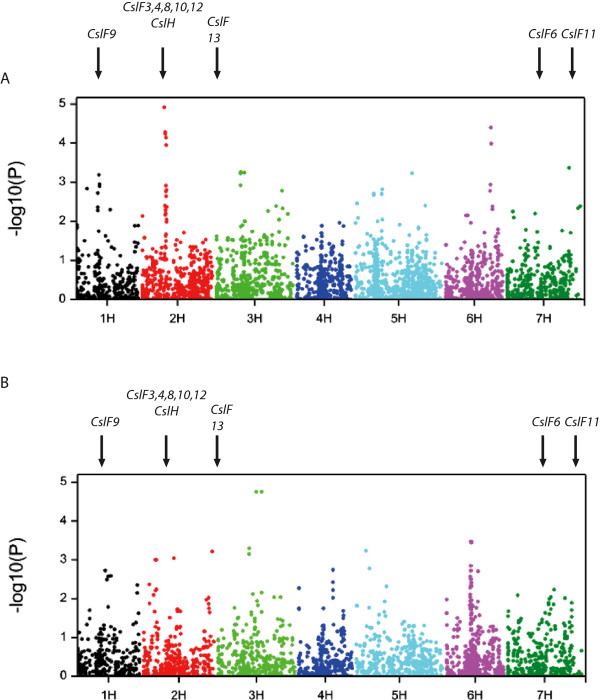
**Manhattan plots of grain (1,3;1,4)-β-glucan content genome wide association scans (GWAS) using the Eigenstrat relationship model.** The -log10 (p-values) from a genome-wide scan are plotted against the position on each of the 7 barley chromosomes. **(A)** Mean Spring grain (1,3;1,4)-β-glucan content. **(B)** Winter grain (1,3;1,4)-β-glucan content. The positions of *CslF9* on 1H, and the *Csl* cluster on 2H, which includes *CslF3, 4, 8, 10* and *12,* are indicated by black downward arrows.

A PCoA showed that the dataset derived from genotyping the elite Spring and Winter barley types using the 9 K SNP iSelect platform [27] could be partitioned by both row type and flowering habit (Figure 1B), with the 2- row Winter and 2- row Spring-types comprising the two larger subgroups. Therefore, we restricted our GWAS to 399 2-row Spring and 204 2-row Winter lines. The PCoA also illustrates that the 2-row Winters are genetically distinct from the 2-row Springs, and STRUCTURE analysis (Figure 1C) revealed residual population structure within these two groups. Consequently we analysed the Winter and Spring accessions separately, and fitted an Eigenstrat model to account for this residual population structure. In total 4,362 SNPs for the Winter- type, and 4,574 SNPs for the Spring type lines distributed across the barley genome satisfied the criteria of having a minimum allele frequency of >0.1 and less than 5% missing data providing an average marker density of approximately 3–4 SNPs/cM. Eigenstrat-adjusted GWAS analyses for (1,3;1,4)-β-glucan content in the grain of the Spring- and Winter–type cultivars are shown in Figure 2 and the corresponding naïve analyses are provided in Additional file 1.

### Associations with genes involved in (1,3;1,4)-β-glucan synthesis and breakdown

We identified 14 significant genome wide associations using an arbitrary threshold of -Log10(P) > 3, with two being found in both populations (named QBgn.SW-2H1 and QBgn.SW-3H1), five specific to the Spring population (QBgn.S-1H1, QBgn.S-3H1, QBgn.S-5H1, QBgn.S-6H1 and QBgn.S-7H1), and seven associations unique to the Winter population (QBgn.W-2H1, QBgn.W-2H2, QBgn.W-2H3, QBgn.W-3H1, QBgn.W-5H1, QBgn.W-5H2, and QBgn.W-6H1) (Table 1, Figure 2,). Associations were found between grain (1,3;1,4)-β-glucan content and genetic loci on all barley chromosomes. We identified associations that coincide with the known position of *CslF9* and *GlbI,* which encodes E1 (a (1–3,1-4)-β-glucanase), on chromosome 1H (QBgn.S-1H1) and the cluster of *CslF* genes on chromosome 2H that includes *CslF8* and *CslH* (QBgn.SW-2H1). After calculating the FDR, two QTLS, QBgn.SW-2H1, and QBgn.W-3H1 had an adjusted p value of <0.025 (Table 1) (<0.015 for both QTLs). Surprisingly, we failed to detect any significant associations around the *CslF6* gene on chromosome 7H which is known to be the primary (1,3;1,4)-β-glucan synthase expressed during grain development. As SNP density on the iSelect platform is relatively low (*ca.* 1 polymorphic SNP every 6 genes) and it does not contain any assays within *CslF6* (MLOC_57200, 72.5 cm), we developed a KASP marker based on a G to A SNP in the third exon of *CslF6* that causes an alanine to threonine substitution (A590T – [29, 30]). Genotyping a selection of individuals chosen to reflect the phenotypic extremes for (1,3;1,4)-β-glucan content revealed that all shared the same allele as *cv.* Morex regardless of grain (1,3;1,4)-β-glucan content, indicating that variation at this nucleotide is not diagnostic for this trait within the elite barley germplasm, in agreement with [29, 30]. This is not entirely surprising as our unpublished data on the three-dimensional model of the HvCslF6 enzyme indicate that the A590T substitution is located far from the active site of the enzyme (JG Schwerdt and GB Fincher, unpublished).

**Table 1:** **Significant (-lod10 p ≥ 3) marker-trait associations identified by genome-wide association scans in elite barley germplasm**

	Peak	Peak (i-select) marker	i-select peak cM	MxBk peak cM	IBSC peak cM	-LOG (10)P	Candidate annotation	Barley gene id/transcript (MLOC)	cM (MxBk)	Morex contig	Car 5 DPA FPKM	Car 15 DPA FPKM	CAZY
**1H**	QBgn.S-1H1	11_11484	51.2	48.4	48.4	3.2	*CslF9*	MLOC_59327	48.1	contig_43489	13.6	9.1	GT2
							*GlbI*	MLOC_62746	54.4	contig_46926	0.5	30.7	GH17
**2H**	QBgn.W-2H1	11_21265	28.4	26.2	n/a	3.0	*HvXTH_5607*	MLOC_5607	23.5	contig_136449	0.2	0.2	GH16
	QBgn.SW-2H1**	11_10498	49.1	50.9	50.9	4.5	*CslF3*	MLOC_59289	55.6	contig_43435	0.1	0.1	GT2
		12_31408	n/a	55.4	53.4	4.9	*CslF4*	-	55.6	contig_6524	0.0	0.0	GT2
							*CslF8*	MLOC_52692	55.6	contig_37718	1.5	1.9	GT2
							*CslF10*	MLOC_13463	55.6	contig_1565725	0.0	0.0	GT2
							*CslF12*	MLOC_7825	55.6	contig_140266	0.1	0.0	GT2
							*CslH*	MLOC_53007	55.6	contig_37984	0.8	1.8	GT2
	QBgn.W-2H2	11_10651	68.2	n/a	62.8	3.0	Glycoside hydrolase, family 9	MLOC_34376	65.4	contig_243681	64.1	30.0	GH9
	QBgn.W-2H3	12_31180	155.3	144.3	n/a	3.2	-	-	-	-	-	-	-
**3H**	QBgn.S-3H1	SCRI_RS_222102	54.8	54.8	54.8	3.3	Glycosyl transferase, family 48	MLOC_501	51.3	contig_103522	7.9	9.9	GT48
							*HvGT48_13377*	MLOC_13377	51.3	contig_1565486	8.4	11.1	GT48
								MLOC_72705	51.6	contig_6218	15.8	11.6	GH1
	QBgn.SW-3H1	SCRI_RS_237939	63.0	63.0	63.7	3.2	*HvGSL7*	MLOC_11267	63.3	contig_1560726	43.3	54.7	GT48
		11_11314	70.2	63.7	61.9	3.3	Glycoside hydrolase, family 5	MLOC_74852	61.9	contig_66652	31.1	4.9	GH5
	QBgn.W-3H1**	SCRI_RS_166119	86.2	86.2	86.3	4.8	Glycoside hydrolase, family 17	MLOC_5621	88.5	contig_136464	15.2	6.5	GH17
		11_20628	98.5	87.4	87.4	4.8	-	-	-	-	-	-	-
**5H**	QBgn.W-5H1	11_21365	21.3	13.1	9.3	3.2	-	-	-	-	-	-	-
	QBgn.W-5H2	SCRI_RS_3280	n/a	93.0	n/a	3.1	*HvCel3*	MLOC_44777	96.3	contig_275346	41.2	9.7	GH9
	QBgn.S-5H1	12_30377	128.7	118.0	118.0	3.2	Cellulose synthase	MLOC_65914	114.0	contig_50865	3.0	0.3	GT2
**6H**	QBgn.W-6H1	SCRI_RS_207174	54.9	54.9	55.2	3.5	Glycoside hydrolase, family 9	MLOC_37664	51.0	contig_2548837	0.3	0.0	GH9
	QBgn.S-6H1	SCRI_RS_189619	102.1	102.1	102.1	4.4	Myb, DNA-binding	MLOC_76165	n/a	contig_70355	0.6	4.4	n/a
**7H**	QBgn.S-7H1	SCRI_RS_230261	140.9	140.9	140.9	3.4	*HvGSL5*	MLOC_64106	140.4	contig_48373	93.4	38.0	GT48
							*GlbII*	MLOC_73214 and MLOC_73215	138.2	contig_6317	N/A	N/A	GT17
							*HvSuSyII*	MLOC_10031	140.6	contig_1558277	10.1	17.7	GT1

Germplasm included in the analysis was a subset of 2-row Spring and Winter accessions from the UK and Northern Europe described in [27]. Peaks with adjusted p values < 0.025 indicated by **. Barley Gene id/transcipt (MLOC), Morex Contig, Developing grain without bracts 5 days post anthesis (CAR 5 DPA FPKM) and CAR 15 DPA FPKM from ([31] IBGS). Barley Gene id/ transcipt (MLOC). FPKM - fragments per kilobase of exon per million fragments mapped. QTL names including S represent associations identified in the Spring population, W in the Winter population and SW in both populations. *EII* on 7H is represented by two gene models and therefore transcript expression data is not available. Dashes represent those regions were no obvious candidate has been identified for that region based on the available annotations.

We then explored whether genes underlying the remaining associations may be responsible for the degradative re-modelling of (1,3;1,4)-β-glucan, or could potentially act as regulatory genes upstream of genes responsible for (1,3;1,4)-β-glucan turnover. The maximum size of the regions associated with grain (1,3;1,4)-β-glucan content varied between 7.1 cM and 15.7 cM (taking into account that we have extended the intervals containing markers with a -Log10(P) > 3 by 2.5 cM from each flanking marker, i.e. 5 cM in total– see Methods). To characterise these genomic regions we extracted the putative gene contents of each interval [31] and compiled a list of potential candidate genes underlying each of the associations. We then restricted this list to genes annotated as putatively involved in complex carbohydrate metabolism and expressed at the transcriptional level (FPKM > 1) in the developing grain according to a deep RNA-seq dataset [31]. This narrowed the set of putative candidate genes considerably (Table 1). Several regions contained glycosyl hydrolase (GH) family members that are known to be capable of hydrolyzing (1,3;1,4)-β-glucan (regions QBgn.S-1H1, QBgn.S-7H1, QBgn.W-2H1, QBgn.W-2H2, QBgn.SW-3H1, QBgn.W-3H1, QBgn.W-5H2, QBgn.W-6H1) or glycosyl transferases (GT) that may modify (1,3;1,4)-β-glucan (QBgn.S-3H1, QBgn.SW-3H1, QBgn.S-7H1)*.* Interestingly, two members of Glycosyl Hydrolase family 17, (1–3,1-4)-β-glucanases that specifically cleave the (1–4)-linkages in (1,3;1,4)-β-glucan (on the reducing side of (1–3)-β-glucosyl residues), and referred to as isozymes EI and EII encoded by *GlbI* and *GlbII* respectively*,* are found within region QBgn.S-1H1 on 1H, collocating with *CslF9,* and within region QBgn.S-7H1 on 7H. Given the surprisingly high frequency of correspondence, we decided to investigate these possible degradative or re-modelling genes further by exploring both their evolutionary and functional relationships.

### Glycosyl Hydrolase (GH) and Glycosyl Transferase (GT) genes expressed during grain development

**Figure 3:**
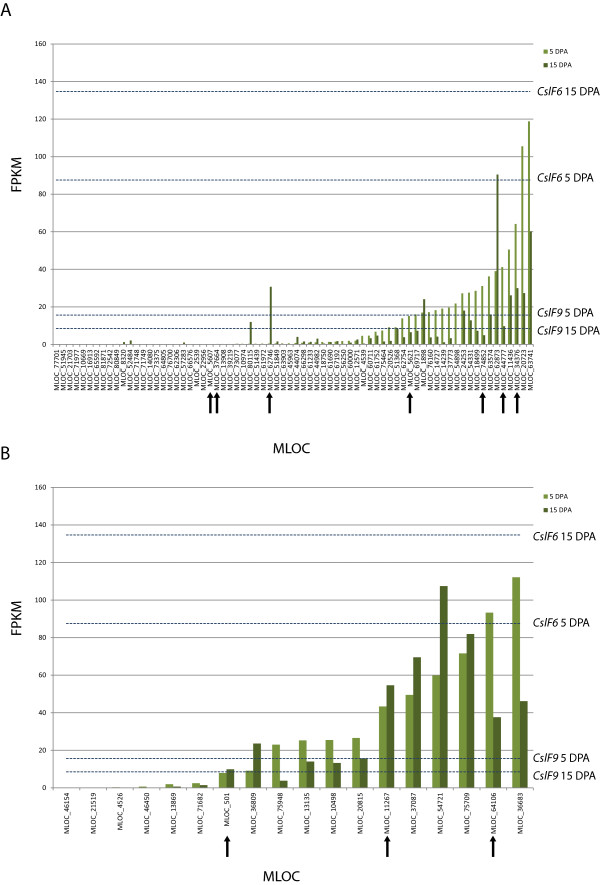
**Expression levels (FPKM) of genes putatively involved in (1,3;1,4)-β-glucan turnover.** This includes two different tissues, 5 days post anthesis caryopsis (5DPA) and 15 DPA caryopsis. Represented are several Glycoside hydrolase (GH) families, of whom at least one member was located in a region identified by GWAS as associated with grain (1,3;1,4)-β-glucan content, GH5, GH9, GH16, and GH17 **(A)**, and genes from Glycosyl transferases family 48 (GT48) **(B)**. Dotted lines representing FPKM values for *CslF6* and *CslF9* at these two time points are included for comparison. Those genes located in regions identified by GWAS as being associated with grain (1,3;1,4)-β-glucan content are highlighted with an arrow.

We first surveyed the available barley genome sequence assemblies [31], using BLAST to catalog putative members of different GH families. After removing those sequences that produced unreliable codon alignments 20 members of family GH1, 10 members of family GH3, 6 members of family GH5, 15 members of family GH9, 18 members of family GH16, and 33 members of family GH17 remained. Phylogenetic analysis using Bayesian inference divided these families into three primary clades (Additional file 2), with all of the GH family members found within regions associated with grain (1,3;1,4)-β-glucan content in the same clade. For all available gene models we then revisited the gene expression atlas [31] to categorise GH gene expression in two grain developmental stages, caryopsis tissue 5 days post anthesis (5 DPA), and caryopsis tissue 15 days post anthesis (15 DPA) (Figure 3). Both of these tissues are characterised by extensive cellular differentiation and wall deposition. At 5 DPA the multi-nucleate coenocytic endosperm is completing the process of rapid cellularisation providing internal structure to the developing grain. This structure continues to develop by cell division and expansion until maximum fresh weight is achieved at around 20 DPA. At 15 DPA the endosperm is entering the so-called ‘soft dough stage’, cell walls are thickening, large starch granules are being deposited and the aleurone cells are clearly differentiated. We therefore predict that the expression of (1,3;1,4)-β-glucan synthases and/or hydrolases during either or both developmental stages would impact final (1,3;1,4)-β-glucan content.

We observed that the expression of *CslF8, CslF9, and CslH* genes was relatively low (1 – 19.9 fragments per kilobase of exon per million fragments mapped (FPKM) in this dataset) at both developmental stages. *CslF6* was moderately expressed at 5DPA (20–49.9 FPKM) and highly expressed (50 – 99.9 FPKM) at 15 DPA. For the 102 glycosyl hydrolase family members, 36 had no detectable expression in either of these two developmental stages (Figure 3). 34 genes showed low levels (1 – 19.9 FPKM) of expression at 5 DPA, and 45 were expressed at this level in the later developmental stage. The expression of these genes is therefore comparable to that of *CslF8, CslF9 and CslH* in these tissues. The remaining genes showed moderate to high levels of expression in both tissues, (21.78 – 3083.35 FPKM) with expression across the two tissues appearing to be generally correlated. We carried out a similar analysis for the barley glycosyl transferase family GT48, identifying 40 putative family members in the barley genome including the seven barley (1,3)-β-glucan synthase-like (*HvGsl*) genes described by [32]. In total 18 genes from this subset could be included in the phylogenetic analysis (Additional file 3). Of these, 14 and 13 were expressed at 5 DPA and 15 DPA, respectively, with six showing high levels of expression at both developmental stages (Figure 3).

We then compared the genetic locations of the GH and GT genes that were expressed at 5DPA and/or 15DPA in developing caryopses with the locations on the barley genome associated with grain (1,3;1,4)-β-glucan content in our GWAS analysis. Eight regions contain members of family GH5 (QBgn.SW-3H1), family GH9 (QBgn.W-2H2, QBgn.W-5H2, and QBgn.W-6H1), family GH16 (QBgn.W-2H1) or family GH17 (QBgn.S-1H1, QBgn.S-7H1 and QBgn.W-3H1). With the exception of MLOC_5607, a GH16 at QBgn.W-2H1, MLOC_37664, a GH9 at QBgn.W-6H1, and *GlbI*, a GH17 at QBgn.S-1H1, all showed moderate levels of expression in both stages of grain development. Expression data was not available for *GlbII,* a GH17, as this gene’s coding sequence (CDS) was split across two gene models. Three regions associated with grain (1,3;1,4)-β-glucan content, (QBgn.S-3H1 and QBgn.SW-3H1 on chromosome 3HL, and QBgn.S-7H1 on chromosome 7HL) contained genes annotated as members of glycosyl transferase family 48. These include *HvGsl5* (QBgn.S-7H1) and *HvGsl7* (QBgn.SW-3H1)*.* All GT48 genes present in regions associated with grain (1,3;1,4)-β-glucan content were expressed in both 5 DPA and 15 DPA caryopses.

## Discussion

GWAS in two collections of elite barley germplasm identified 14 significant associations (Log10(P) > 3) between SNP markers and grain (1,3;1,4)-β-glucan content, with seven occurring in the Spring and nine in the Winter population. Two of these associations, QBgn.SW-2H1 which collocates with the cluster of *Csl* genes on 2H, and QBgn.SW-3H1 on the long arm of chromosome 3H, were effectively cross-validated as they were detected in both the Spring and Winter genepools. QBgn.SW-3H1 was also identified in a recent GWAS of grain (1,3;1,4)-β-glucan content in the North American Barley Coordinated Agricultural Project (CAP) germplasm [33]. The identification of associations in intervals that coincide with the known positions of the cluster of *CslF* genes on chromosome 2H and the region on chromosome 1H which includes *CslF9* and *Glb1,* genes known to be capable of synthesising and breaking down (1,3;1,4)-β-glucan [12, 34] served to validate our GWAS approach, while the 12 other associations provide interesting new avenues to further our understanding of the synthesis and regulation of grain (1,3;1,4)-β-glucan. Further confidence in these associations is provided due to previous expression analyses of *CslF* and *CslH* gene family members by qRT-PCR, which identified *CslF6* and *CslF9* as the major (1,3;1,4)-β-glucan synthases expressed in various stages of barley grain development, with *CslF8* and *CslH* also expressed in this tissue but at very much lower levels [31, 34]. In addition to the *CslF* genes on 1H and 2H, several of the associations identified by GWAS occur in the same region as QTL described in previous studies [11, 22, 33–36]. However, many others appear to be novel.

GWAS is often credited with providing greater genetic resolution of regions associated with a trait of interest [37–39] compared to bi-parental mapping approaches and here we were able to confirm and refine several previously identified QTLs. Using a threshold of - log10 p ≥ 3 we were able to attribute many of these relatively narrow QTLs to locations that had previously been found to underly a QTL for the same trait [11, 22, 33–36] or genes know to influence grain (1,3;1,4) β- glucan content [12, 34]. We also applied a 5% FDR, after which only two QTL retained a highly significant adjusted p-value. However given the degree of QTL cross-validation with independent datasets we suggest this FDR is overly stringent for our dataset. Li et al. [35] used a bi-parental population derived from CDC bold and TR251 to identify seven QTLs associated with grain (1,3;1,4)-β-glucan content. When the centimorgan positions of the map used in [35] were converted to the MxB map positions [31] (see Methods), QTL3H.2 appears to coincide with association peaks QBgn.S-3H1 and QBgn.SW-3H1 in the current study. The same authors noted that their QTL2.1 and 7.1 were likely the same as QTL identified on corresponding chromosomes by both [11] in a cross between Steptoe and Morex and [22] in a cross between Beka and Logan. While neither group was able to identify the gene(s) underlying these QTL, the recent publication of the barley genome assembly [31] allows us to make such predictions. Thus, these QTL almost certainly coincide with the location of the chromosome 2H *CslF* gene cluster identified in both the Winter and Spring genepools in our analysis, and with the location of the *CslF6* gene on chromosome 7H. Furthermore, two of the three grain (1,3;1,4)-β-glucan content QTL identified by Szucs et al. [36] in the Oregon Wolf Barley (OWB) mapping population were similarly identified here as *CslF9* on chromosome 1H in the Spring germplasm and the *Csl* cluster on chromosome 2H. Islamovic et al. [40] identified a QTL for grain (1,3;1,4)-β-glucan content at between 54.0 – 57.2 cM on chromosome 6H in a population derived from Falcon and Azul, which have moderate and high grain (1,3;1,4)-β-glucan content levels respectively. This appears to correspond to the location of association peak QBgn.W-6H1 on chromosome 6H in the Winter genepool. Based on annotations from both barley and rice we observed that this region contains a family GH9 enzyme and several transcription factors, which could plausibly regulate (1,3;1,4)-β-glucan synthases or hydrolases. Finally, the region underlying QBgn.S-7H1 on the long arm of chromosome 7H contained several potential candidate genes based on their annotations. Particularly intriguing was *Sucrose Synthase II* (*HvSuSyII*) as it has been previously shown that *SuSy* supplies UDP-Glc, the substrate required for the synthesis of cellulose [41], (1,3;1,4)-β-glucans and (1,3)-β-glucans. It has been proposed that *SuSy* could be responsible for channelling UDP-Glc into (1,3;1,4)-β-glucan synthesis [42]. However, as Urbanowicz et al. [43] were unable to demonstrate this relationship *in vitro*, the role of *SuSy* in (1,3;1,4)-β-glucan synthesis remains to be conclusively proven. Alternatively the association between (1,3;1,4)-β-glucan levels and *SuSy* may be related to carbon partitioning between (1,3;1,4)-β-glucan and starch [19].

We were initially somewhat surprised that we failed to identify an association with variation in *CslF6* as it is well-established as the primary enzyme involved in determining grain (1,3;1,4)-β-glucan biosynthesis, based on the observation that *CslF6* knockout mutations effectively contain no grain (1,3;1,4)-β-glucan [30]. A QTL for grain (1,3;1,4)-β-glucan on 7H attributed to variation in *CslF6* has been reported in several mapping populations [11, 29, 35]. However in several other studies using different mapping populations this QTL has not been observed [40, 44, 45]. A survey of regional haplotype diversity across this locus in our association panels indicates that our failure to detect an association may be either because the region is nearing genetic fixation in our elite germplasm, or that effective alleles are present at a low frequency (i.e. below our 10% MAF cut-off). A further explanation could be that we may simply not have had a sufficiently informative and tightly linked SNP marker on our genotyping array. The latter seems unlikely as a KASP assay for *CslF6* based on a non-synonymous nucleotide substitution showed no association with (1,3;1,4)-β-glucan content. As in many other studies this highlights the difficulty in identifying diagnostic genetic markers that are in tight linkage disequilibrium with natural variants of genes underlying quantitative traits. Despite all we know about *CslF6*, at this time only induced mutations have been shown to have a direct to impact on the (1,3;1,4)-β-glucan content of barley grain [30, 46].

We failed to detect at least two other loci that have been implicated in grain (1,3;1,4)-β-glucan content from recent genetic studies. Mezaka et al. [47] mapped (1,3;1,4)-β-glucan content close to the *NAKED* (*NUD*) locus using GWAS on a small population of lines and the SNP genotyping platform described in [48]. It is not known if *NUD* directly influences (1,3;1,4)-β-glucan content, however it is possible that the absence of the hull, which will have a very low (1,3;1,4)-β-glucan content, will result in an overall apparent increase in (1,3;1,4)-β-glucan in hulless varieties. No hulless varieties were included in our grain (1,3;1,4)-β-glucan content analysis. Finally, Chutimanitsakun et al. [49], reported a QTL for grain (1,3;1,4)-β-glucan content associated with *granule-bound starch synthase I* (*GBSS1*) at the *Waxy* (*WX*) locus on the short arm of chromosome 7H (12.5 cM). While the precise mechanism for this remains unknown, perturbations in starch metabolism have been shown previously to affect grain (1,3;1,4)-β-glucan content [50] and this observation may represent another natural example of the regulatory link between starch and (1,3;1,4)-β-glucan metabolism [19].

While the hypothesis that grain (1,3;1,4)-β-glucan content is determined by genes that synthesise (1,3;1,4)-β-glucan is persuasive, it is equally valid that allelic variation in genes involved in the breakdown or re-modelling of (1,3;1,4)-β-glucan could also be responsible. Based on the combination of genetic co-location and tissue specific gene expression, the current work hints at the potential importance of β-glucan endohydrolases in (1,3;1,4)-β-glucan turnover and thus in determining final grain (1,3;1,4)-β-glucan content [11, 51–53]. Indeed, it is becoming apparent that during the biosynthesis of many cell wall polysaccharides by polysaccharide synthases, genes encoding enzymes capable of hydrolysing the nascent polysaccharide are also expressed [54]. Whether the hydrolases function to release chains from the synthase enzyme, or somehow trim the newly synthesised chains, is not yet clear [54]. In particular, we considered it interesting that QBgn.S-1H1 on 1H, and QBgn.S-7H1 on 7H, coincide with the known location of *GlbI* and *GlbII* respectively. These encode (1,3;1,4)-β-glucan-specific family GH17 (1,3;1,4)-β-glucanases known as isoenzymes EI and EII [55]. The commonly known role of these enzymes is to hydrolyse (1,3;1,4)-β-glucan [56], however it has also been suggested that (1,3;1,4)-β-glucanases are involved in (1,3;1,4)-β-glucan synthesis [34] providing a means of editing [57] or dispensing the completed polysaccharide chains after assembly [58]. The family GH5 gene on chromosomes 3H and the family GH9 genes on chromosomes 2H, 5H and 6H are (1,4)-β-glucanases that can hydrolyse (1,4)-β-glycosyl linkages in a range of polysaccharides, including cellulose, (1,3;1,4)-β-glucans, (1,4)-β-xylans and xyloglucans [59]. However, (1,3;1,4)-β-glucans are not the preferred substrates for these enzymes and it is not clear whether they are likely to be involved in (1,3;1,4)-β-glucan metabolism in barley. Similarly, the family GH17 gene on chromosome 3H (Table 1), which has the highest LOD score for a hydrolase and encodes a (1,3)-β-glucanase designated isoenzyme GIII [60] will not hydrolyse (1,3;1,4)-β-glucans and is more likely to function in the removal of callose or in response to a biotic or abiotic stress. At this stage it is difficult to envision how a (1,3)-β-glucanase might be involved in (1,3;1,4)-β-glucan synthesis or regulation.

The association with the family GH16 gene on chromosome 2H is of special interest. This gene encodes an enzyme designated as a xyloglucan xyloglucosyltransferase or xyloglucan endo-transglycosylase (XET). Most of the enzymes in the GH16 family are of microbial origin, except for the xyloglucan endo-transglycosylases (XETs), which are widely distributed in higher plants [59]. The XETs are known to modify cell wall xyloglucans [61] but can also catalyze transglycosylation reactions involving (1,3;1,4)-β-glucans [53]. Despite the fact that barley grain has extremely low levels of xyloglucan, this gene is expressed at relatively high levels in 5 and 15DAP caryopses. It has been proposed that the assembly of (1,3;1,4)-β-glucans requires the action of multiple enzymes [62] in addition to the CslF’s and CslH, and other enzymes implicated in the process include the XETs from family GH16 [53, 54, 62, 63]. Thus the GWAS analysis reported here could provide additional, non-biased evidence for a possible role for XETs in (1,3;1,4)-β-glucan synthesis (Table 1).

The GWAS also revealed associations between grain (1,3;1,4)-β-glucan levels and regions of the genome where family GT48 glycosyl transferase genes are located. The GT48 genes are variously designated as *callose synthase genes* (*CalS*) or *glucan synthase-like genes* (*GSL*). Callose is a (1,3)-β-glucan that is deposited in specialized tissues and cells, such as pollen mother cell walls, pollen tubes, in abscission zones, on sieve plates in phloem of dormant plants, in plasmodesmatal canals and at wound sites [64]. Although some concerns are still raised about the precise role of GSL proteins in higher plants, the balance of evidence would support their participation in (1,3)-β-glucan synthesis [64]. As noted previously, Burton et al. [62] suggested that (1,3;1,4)-β-glucans might be assembled in a two phase process that involves more than one enzyme, and it was proposed that either XETs or GT48 callose synthases might be involved. We identified strong associations between (1,3;1,4)-β-glucan levels and both an XET and a GT48 (1,3)-β-glucan synthase. The roles of these genes can now be tested as potential determinants of the levels and fine structures of (1,3;1,4)-β-glucans in barley grain.

## Conclusion

Premium end users of barley have particular specifications for grain characteristics such as nitrogen content [65], grain size [66], and alpha amylase content [67]. In the malting and brewing industries they also require low (1,3;1,4)-β-glucan, but in future applications in human health and nutrition, high levels of (1,3;1,4)-β-glucans are likely to become desirable. The malting and brewing preferences will have shaped the genetic variation present in the elite barley germplasm exploited in our analysis. They will also have largely determined the associations that we identified. Pauly and Keegstra, [68] discussed the difficulties and complexities of manipulating plant cell wall composition by up- or down-regulating genes known to be involved in the synthesis and degradation of cell wall polymers. Despite the observation that single CslF6 mutants have no grain (1,3;1,4)-β-glucan suggesting simple genetic control, the mutant plants express a range of defects that indicate an overall lack of fitness [30]. This is consistent with both the importance of (1,3;1,4)-β-glucan in barley growth and development and our hypothesis that natural variation in (1,3;1,4)-β-glucan content is the product of a complex regulatory interaction between genes involved in carbohydrate polymer synthesis, re-modelling and breakdown. Different suites of genes may be involved in different tissues/cell types, and there is good evidence for an overarching environmental component. While this clearly adds complexity, using contemporary genetics to identify the genes (or markers) that contribute to the phenotype will ultimately be useful in tracking and selecting high or low (1,3;1,4)-β-glucan lines in barley improvement programs.

## Methods

### Genetic materials and growth conditons

A collection of 399 elite 2-row Spring-type, and 204 elite 2-row Winter- type barley cultivars were grown and phenotyped for grain (1,3;1,4)-β-glucan content. For the Spring barleys, two single plant replicates were grown in 25 cm pots in a polytunnel in Dundee in corresponding spatial row-column design with replicate blocks. During the growing season, plants were given a single fungicide treatment, which was sufficient to maintain good plant health for the entire season. For the Winter cultivars a single replicate was grown at Balruddery farm, Scotland, in 2012 and 2013. All of the grain was sampled from individual plants by mechanical threshing and stored until processed for (1,3;1,4)-β-glucan content.

### Genotypic information

All plants were genotyped by standard approaches using a 9 K barley iSelect SNP genotyping platform described previously [27]. For initial genotype calling, the automated cluster algorithm GenTrain 2.0 was applied. Prior to GWAS, markers with a minimum allele frequency of less than 10% and those that had >5% missing data points were removed from the data matrix.

### Quantification of (1,3;1,4)-β-glucan content

The concentration of (1,3;1,4)-β-glucan in barley flour was determined using a modified version of the Megazyme (1,3;1,4)-β-glucan assay, based on the “Streamlined method” (McCleary method; AOAC Method 995.16, AACC Method 32–23, ICC Standard Method No. 168). Glucose oxidase/peroxidise (GOPOD) reagent, lichenase and β-glucosidase enzymes were purchased from Megazyme Int., Wicklow, Ireland. All flour samples were prepared in a Powerlyser™ ball-bearing grinder (MO BIO, CA, USA). A total of 10 grains per sample were milled for up to 5 min each to a consistent fine powder. Two technical replicates were performed on all samples. Samples (15 mg) were weighed into 2 ml Eppendorf tubes and 1 ml sodium phosphate buffer (20 mM, pH 6.5) added. Samples were mixed well and placed in an Eppendorf Thermomixer Comfort at 25°C and ramped up to 90°C over a 30 min period with mixing at 1000 rpm (i.e. ramping of 2°C/min from 25°C to 90°C, followed by 15 min at 90°C). Samples were allowed to cool to 50°C before incubation at 50°C with 40ul lichenase enzyme (50 U/ml in 20 mM sodium phosphate buffer, pH6.5) in the Thermomixer with mixing at 1000 rpm for 1.5 hr. The enzymic reaction was stopped with the addition of 0.8 ml sodium acetate buffer (200 mM, pH 4.0). Samples were equilibrated to room temperature for approximately 10 min, allowing particulate matter to settle before centrifugation at 10,000 rpm for 10 min. Two 50ul aliquots of supernatant were reacted to completion at 50°C with 50ul glucosidase enzyme (2U/ml in 200 mM acetate buffer) and a single aliquot was incubated with 50ul sodium acetate buffer (200 mM, pH 4.0) as a reagent blank in a 2 ml × 96 well deep-well plate in the Thermomixer for 20 min. GOPOD reagent (1.5 ml) was added to each well and incubated in the Thermomixer at 50°C for 30 min. Aliquots (200μl) were transferred to a 96 well flat-bottomed micro-plate and read on a plate reader (Thermo Multiskan spectrum) at 510 nm. With each set of determinations, glucose (50 ug), a water control and two flour standards (2 replicates) were included. (1,3;1,4)-β-Glucan content was adjusted to these standards providing the standards value was between 4.05% and 4.15%, otherwise the batch was repeated. (1,3;1,4)-β-Glucan content calculations were carried out exactly as detailed in the Megazyme kit.

### Statistical analysis and GWAS

Simple linear regression analysis was carried out by use of GenStat version 15 to evaluate contributions of variation between replicates and years, to phenotypic variation in (1,3;1,4)-β-glucan content in both Winter and Spring populations. Analysis of variance was carried out to identify the source of variation in grain (1,3;1,4)-β-glucan in this dataset. To identify population structure within our dataset we used GenALEx to carry out a principal coordinate’s analysis (PCoA) using data from 766 elite barley accessions, including both 2-row and 6-row, Winter and Spring germplasm (Additional file 4), which had been genotyped using the 9 K SNP iSelect platform [27]. We then used the Bayesian clustering program STRUCTURE version 2.3.4, selecting an admixture model with correlated allele frequencies, for the number of populations (k) = 4 (ten replicates), with a burn-in period of 10 × 10^3^ iterations followed by 10 × 10^3^ MCMC iterations [69]. For both the Winter and Spring lines the GWAS was carried out in GenStat version 15 using the Eigenanalysis relationship model with SNP map positions as per [27]. In each case we ran a naïve model for comparison. The GWAS was carried out on a subset of the 2 row lines for each flowering habit, 399 Spring- type barley, and 204 Winter- type barleys, for which we assayed for grain (1,3;1,4)-β-Glucan content (Additional file 4). For the spring barley analysis the mean (1,3;1,4)-β-glucan content from the two biological replicates was used, while the two years of data collected from the Winter lines were analysed together, considering each year as an environment. Significant SNPs positioned within 5 cM of each other were considered to be linked to the same QTL, with the more significant chosen as representing the QTL. Nomenclature of QTLs, and use of the trait abbreviation, Bgn, in these QTL names follows the system described in [36] and OWB-DGGT (http://wheat.pw.usda.gov/ggpages/maps/OWB/). A stringent false discovery rate (FDR) < 5% was calculated using the qvalue package [70] in R version 3.1.1 (R core team 2014) to provide adjusted p values. *CslF and CslH* genes were mapped onto the barley genetic map relative to the location of SNPs used for GWAS using a combination of Barley Genome Zippers [71] and a sequence assembly of the cv. Morex Genome (Morex V.3.0 [31]). These resources were used in combination with http://floresta.eead.csic.es/barleymap/ to provide annotations for genes within the intervals identified by the association analysis. When querying http://floresta.eead.csic.es/barleymap/ we extended the interval by 2.5 cM to take account of map order uncertainty. Where map positions differed between resources (SNP iSelect platform position [27], Morex x Barke map from the Genome Zippers, [71], and the Morex genome assembly [31]), we preferred those from the Morex x Barke map. This is because these markers have been used to genetically anchor the physical map of barley, whereas the Morex genome assembly includes this information plus marker positions derived from synteny and linkage disequilibrium (LD). We queried the RNA-seq based gene expression atlas developed as part of the barley genome assembly [31] to provide data on gene expression for those genes designated as candidates based on their annotations. We mined this resource for two developmental stages; 5 days post anthesis (5 DPA) caryopsis, and 15 DPA caryopsis.

### Phylogenetic analysis of barley glycoside hydrolases and glycosyl transferases

Coding sequences for members of 13 glycoside hydrolase (GH) families previously shown to be involved in the breakdown of either (1,3)- or (1,4)-β-glycosidic linkages [59] were identified based on their annotation using the MIPs FTP site [31]. A similar analysis was carried out for glycosyl transferase family 48 (GT48). Sequences were aligned in MEGA version 5.2.2 [72] to produce a codon based alignment using the MUSCLE algorithm. Bayesian phylogenetic analysis was carried out on these two subsets of sequences as described in [14] using Block Mapping and Gathering with Entropy analysis (BMGE) [73] and TOPALi V2.5 [74].

### KASP genotyping

An allele specific assay was designed with the KASP By Design system based on a SNP described in [30] and [29] in the third exon of the *CslF6* gene (Additional file 5) and used to genotype a set of accessions with divergent grain (1,3;1,4)-β-glucan contents (Table 2). KASP By Design assay reactions were carried out using a 8 μl reaction mix containing 20 ng DNA, 2× KASPar v4.0 Reagent (KBS -1016) and 0.11 μl KASP By Design –Non Validated SNP assay (KBS -1013). PCR was performed on StepOnePlus using the following program; 20°C, 2 min pre-PCR read; 94°C, 15 min, 10 cycles (94°C, 20 sec; 62°C, 1 min, decreasing by 0.7°C per cycle); 32 cycles (94°C, 20 sec, 55°C 1 min) 20°C, 2 min post-PCR read. The analysis was performed using default parameters on the StepOnePlus.

**Table 2:** **KASP genotyping results for elite accessions representing extremes of phenotype observed in the germplasm**

Cultivar	Habit	Grain ***(1,3;1,4)-β-glucan***	***CslF6***Exon (A590T)
Duet	Winter	Low	**G**
Kaskade	Winter	Low	**G**
Chicane	Winter	Low	**G**
Magie	Winter	Low	**G**
Kingston	Winter	Low	**G**
Sombrero	Winter	Low	**G**
Baraka	Winter	Low	**G**
Wintmalt	Winter	Low	**G**
Louise	Winter	High	**G**
Frolic	Winter	High	**G**
Winner	Winter	High	**G**
Puffin	Winter	High	**G**
Vesuvius	Winter	High	**G**
Diadem	Winter	High	**G**
Sevilla	Winter	High	**G**
Cobalt	Winter	High	**G**
Aspen	Spring	Low	**G**
Nimbus	Spring	Low	**G**
Lithium	Spring	Low	**G**
Rakaia	Spring	Low	**G**
Appaloose	Spring	Low	**G**
Skittle	Spring	Low	**G**
Dallas	Spring	Low	**G**
Chariot	Spring	Low	**G**
Pongo	Spring	High	**G**
Primera	Spring	High	**G**
Kenia	Spring	High	**G**
Betzes	Spring	High	**G**
Hart	Spring	High	**G**
Isaria	Spring	High	**G**
Century	Spring	High	**G**
Gull	Spring	High	**G**

## Electronic supplementary material

Additional file 1: **Manhattan plots of grain (1,3;1,4)-β-glucan content genome wide association scans (GWAS) using the naïve model.** The -log10 (p-values) from a genome-wide scan are plotted against the position on each of the seven barley chromosomes. (A) Mean Spring grain (1,3;1,4)-β-glucan content. (B) Winter grain (1,3;1,4)-β-glucan content. The positions of *CslF9* on 1H, and the *Csl* cluster on 2H, which includes *CslF3, 4, 8, 10, 12* and CslH*,* are indicated by black downward arrows. (PDF 314 KB)

Additional file 2: **An unrooted bayesian tree of Glycoside hydrolase (GH) families putatively involved in (1,3;1,4)-β-glucan turnover.** Genes/transcripts identified as candidates in the current association study are highlighted in bold and in larger font than other genes. Posterior probabilities are provided on branches and a codon position model was used to construct the tree. GH family assignments based on http://www.cazy.org/ [59] are colour coded by family; GH1 = purple, GH3 = blue, GH5 = green, GH9 = orange, GH16 = black, GH17 = brown. (PDF 572 KB)

Additional file 3: **An unrooted bayesian tree of Glycosyl transferases family 48 (GT48).** Posterior probabilities are provided on branches and a codon position model was used to construct the tree. Genes/transcripts identified as candidate genes in the current association study are highlighted in bold and in larger font than other genes. (PDF 350 KB)

Additional file 4: **List of germplasm used in GWAS and (1,3;1,4)-β-glucan content for those accessions assayed.** (XLSX 40 KB)

Additional file 5: **Sequence information of KASP genotyping assay designed to** ***CslF6***. (XLSX 12 KB)
