# Supplementary figures and images for: A genome wide association scan for (1,3;1,4)-β-glucan content in the grain of contemporary 2-row Spring and Winter barleys
Source: BMC Genomics. 2014 Oct 17;15(1):907. doi: 10.1186/1471-2164-15-907 (PMC4213503; doi:10.1186/1471-2164-15-907)

A

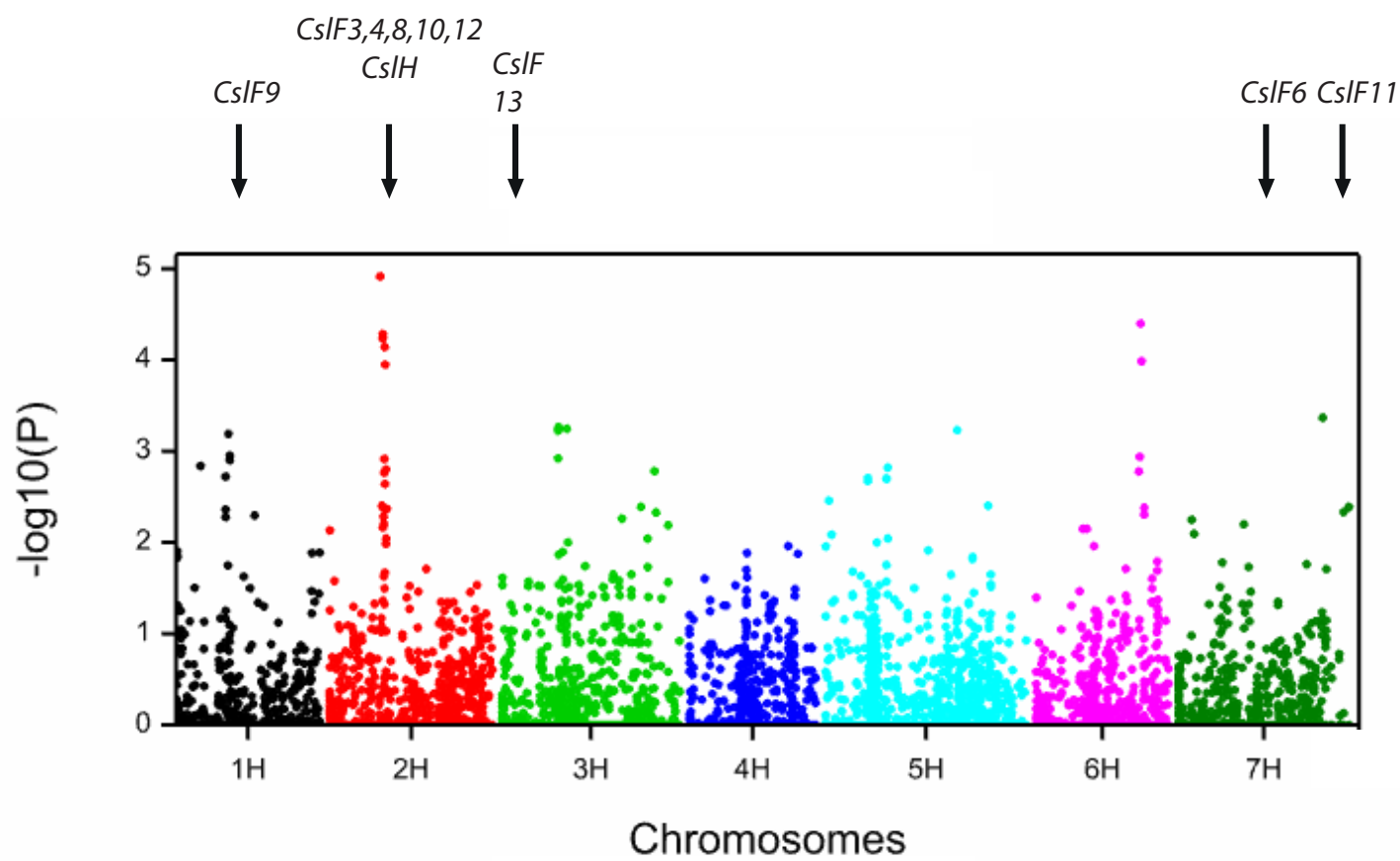

B

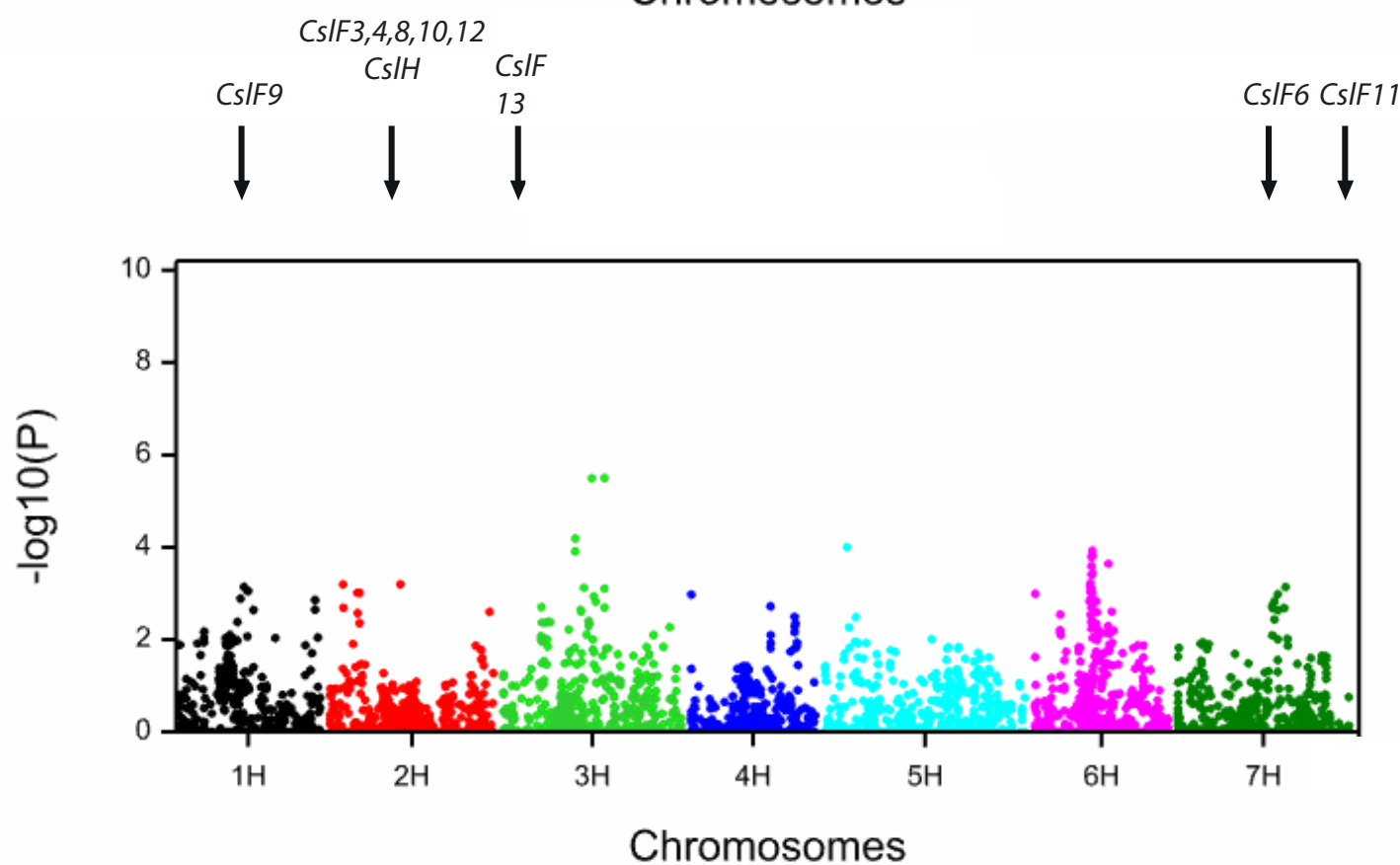

Supplement: Supplementary file 1 — Additional file 1: Manhattan plots of grain (1,3;1,4)-β-glucan content genome wide association scans (GWAS) using the naïve model. The -log10 (p-values) from a genome-wide scan are plotted against the position on each of the seven barley chromosomes. (A) Mean Spring grain (1,3;1,4)-β-glucan content. (B) Winter grain (1,3;1,4)-β-glucan content. The positions of CslF9 on 1H, and the Csl cluster on 2H, which includes CslF3, 4, 8, 10, 12 and CslH, are indicated by black downward arrows. (PDF 314 KB) [file 12864_2014_6608_MOESM1_ESM.pdf]

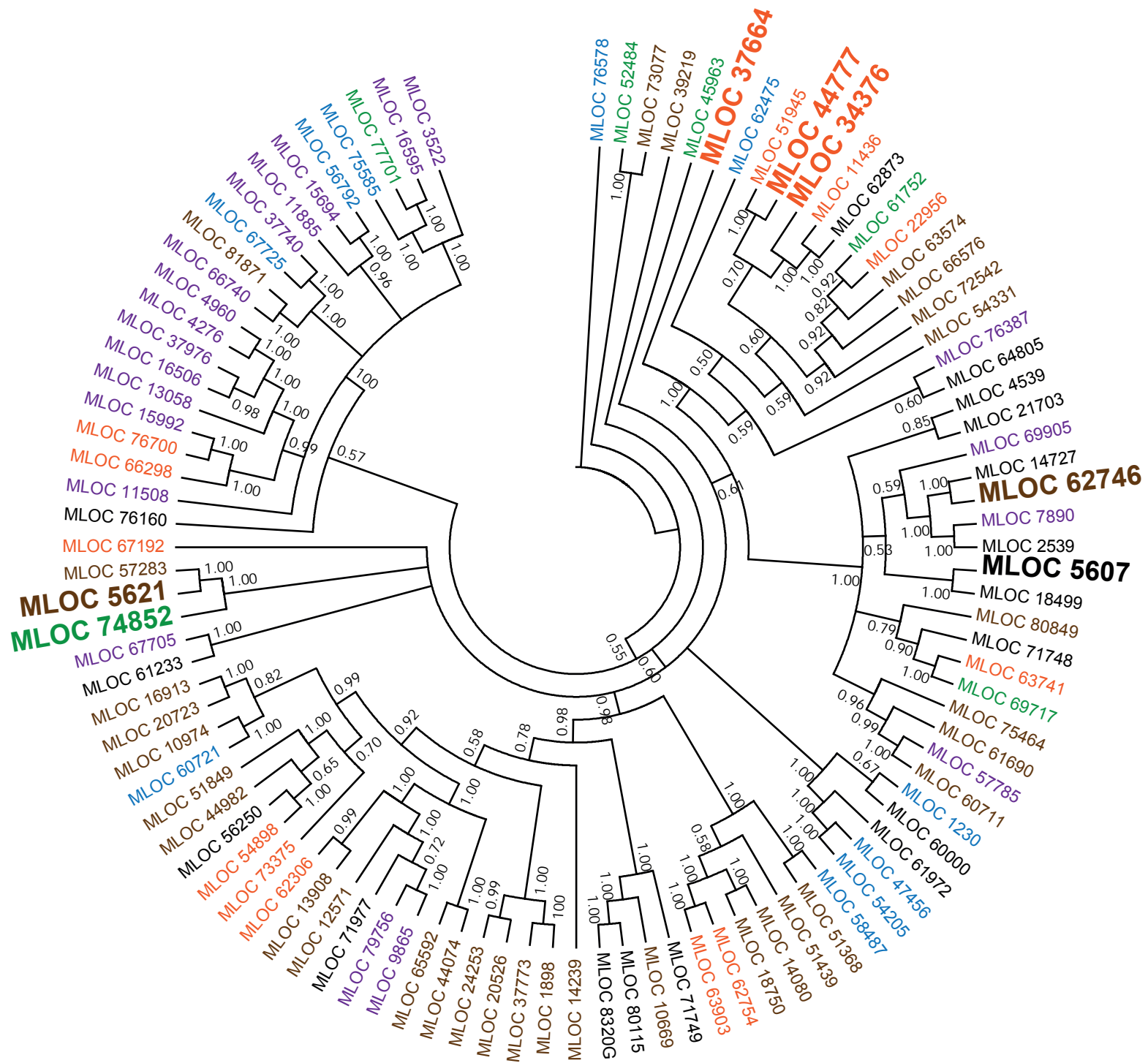

Supplement: Supplementary file 2 — Additional file 2: An unrooted bayesian tree of Glycoside hydrolase (GH) families putatively involved in (1,3;1,4)-β-glucan turnover. Genes/transcripts identified as candidates in the current association study are highlighted in bold and in larger font than other genes. Posterior probabilities are provided on branches and a codon position model was used to construct the tree. GH family assignments based on http://www.cazy.org/ [59] are colour coded by family; GH1 = purple, GH3 = blue, GH5 = green, GH9 = orange, GH16 = black, GH17 = brown. (PDF 572 KB) [file 12864_2014_6608_MOESM2_ESM.pdf]

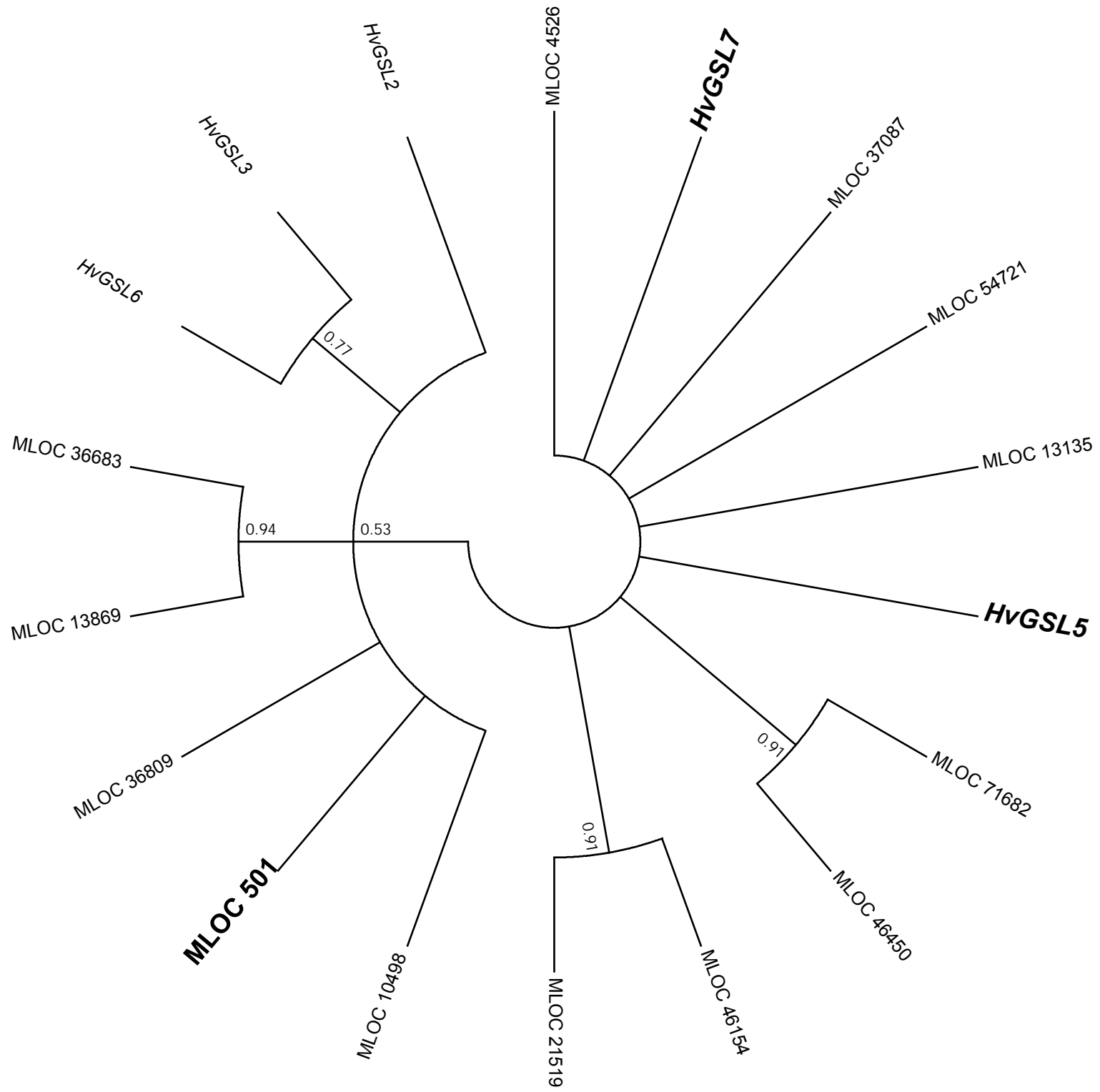

Supplement: Supplementary file 3 — Additional file 3: An unrooted bayesian tree of Glycosyl transferases family 48 (GT48). Posterior probabilities are provided on branches and a codon position model was used to construct the tree. Genes/transcripts identified as candidate genes in the current association study are highlighted in bold and in larger font than other genes. (PDF 350 KB) [file 12864_2014_6608_MOESM3_ESM.pdf]
